# Progressive Supranuclear Palsy Unmasked After Post-COVID-19 Functional Decline in an Elderly Patient: A Diagnostic Challenge

**DOI:** 10.7759/cureus.109779

**Published:** 2026-05-27

**Authors:** Enrico Fulco

**Affiliations:** 1 Department of Medical and Surgical Sciences, Alma Mater Studiorum University of Bologna, Bologna, ITA; 2 Department of Primary Health Care, Internal Medicine Unit addressed to Frailty and Aging, Ospedale Santa Maria delle Croci, Ravenna, ITA

**Keywords:** dat-spect, neuroimaging, parkinsonism, progressive supranuclear palsy, sars-cov-2

## Abstract

Progressive supranuclear palsy (PSP) is a rare neurodegenerative tauopathy characterized by early postural instability, vertical supranuclear gaze palsy, axial rigidity, and poor response to levodopa therapy. Diagnosis remains challenging, particularly in the early stages, because of overlap with other Parkinsonian syndromes. We report the case of an 83-year-old woman who developed progressive gait instability, severe functional decline, dysphagia, and marked weight loss following a mild SARS-CoV-2 infection. Extensive investigations initially excluded metabolic, infectious, neoplastic, and structural causes. Neurological examination revealed axial and limb bradykinesia, hypomimia, hypophonia, apraxia, severe postural instability, and vertical gaze limitation. Oculomotor assessment also documented square wave jerks and eyelid opening apraxia. Levodopa therapy was ineffective. Brain MRI demonstrated marked midbrain atrophy with relative preservation of pontine volume, producing the characteristic hummingbird sign and Mickey Mouse sign. DAT-SPECT showed bilateral putaminal dopaminergic deficit, supporting a degenerative Parkinsonian syndrome. A diagnosis of probable PSP was established according to Movement Disorder Society criteria, clinical presentation, absence of levodopa responsiveness, and supportive imaging findings. This case highlights the diagnostic complexity of PSP when nonspecific systemic manifestations precede neurological deterioration and emphasizes the value of integrating clinical assessment with structural and functional neuroimaging.

## Introduction

Progressive supranuclear palsy (PSP) is an atypical Parkinsonian syndrome belonging to the group of 4-repeat tauopathies. It is characterized by early postural instability, vertical supranuclear gaze palsy, axial rigidity, and cognitive and behavioral changes. The prevalence is estimated at 5-7 per 100,000 individuals and increases with age [1].

Recent advances in diagnostic criteria have improved recognition of the clinical heterogeneity of PSP, including the classical Richardson syndrome phenotype and several atypical variants such as PSP-Parkinsonism, PSP with predominant gait freezing, and corticobasal syndrome presentations [1,2]. Early diagnosis remains difficult because initial manifestations may overlap with Parkinson’s disease, multiple system atrophy, corticobasal degeneration, and vascular Parkinsonism. Neuroimaging plays an increasingly important role in supporting diagnosis, particularly through the identification of characteristic MRI findings such as selective midbrain atrophy and functional imaging evidence of presynaptic dopaminergic degeneration [3,4].

MRI findings typically include midbrain atrophy with relative preservation of the pons, producing the hummingbird sign on sagittal images and the Mickey Mouse sign on axial sections [3,5]. DAT-SPECT demonstrates presynaptic nigrostriatal degeneration and may help distinguish degenerative from non-degenerative causes of Parkinsonism, although findings may overlap among atypical Parkinsonian syndromes [4].

In addition, systemic infections and inflammatory stressors have been hypothesized to precipitate clinical decompensation in patients with underlying neurodegenerative vulnerability [6,7]. We present the case of an elderly woman who developed progressive functional and neurological decline following SARS-CoV-2 infection and was subsequently diagnosed with probable PSP.

## Case presentation

An 83-year-old woman was admitted to the Emergency Department in November 2021 because of progressive gait disturbance associated with inability to ambulate, which had developed over approximately two weeks.

Her past medical history was unremarkable, with no known neurological or systemic chronic diseases. In July 2021, she experienced a mild SARS-CoV-2 infection with a paucisymptomatic course and complete apparent recovery. In the following months, she progressively developed anorexia, worsening dysphagia, and significant involuntary weight loss of approximately 20 kg without an identifiable organic cause at the initial evaluation.

Given the systemic manifestations, she underwent extensive otolaryngological, endoscopic, and radiological investigations, which excluded neoplastic, structural, and metabolic causes.

Neurological examination revealed a complex Parkinsonian syndrome characterized by axial and limb bradykinesia, mild axial rigidity, hypomimia, hypophonia, limb apraxia, severe postural instability, and marked gait impairment with reduced stride length and impaired turning. Postural reflexes were severely impaired, and the patient was unable to ambulate independently. Oculomotor examination demonstrated slowing and limitation of vertical saccades with supranuclear vertical gaze palsy, while vestibulo-ocular reflexes were relatively preserved. Horizontal eye movements were mildly slowed. Square wave jerks were observed during fixation. Eyelid opening apraxia was present, with the patient exhibiting involuntary eyelid closure not attributable to blepharospasm. No definite frontal release signs were elicited. Formal neuropsychological testing was not performed because of the patient’s clinical condition.

Levodopa therapy was initiated at a dosage of 100/25 mg three times daily and progressively escalated to 600 mg daily over two weeks. Treatment response was assessed clinically through repeated neurological examinations focusing on bradykinesia, gait impairment, postural stability, and functional mobility. No meaningful clinical improvement was observed despite dose escalation, consistent with poor levodopa responsiveness and atypical Parkinsonism.

Routine laboratory investigations performed on admission were entirely within normal limits, with no evidence of inflammatory, infectious, metabolic, or endocrine abnormalities (Table 1).

**Table 1:** Laboratory findings on admission Routine laboratory investigations on admission were within normal limits, with no evidence of metabolic, infectious, inflammatory, or endocrine abnormalities that could explain the patient’s neurological deterioration. AST: Aspartate Aminotransferase, ALT: Alanine Aminotransferase, ALP: Alkaline Phosphatase, ESR: Erythrocyte Sedimentation Rate, HbA1C: Hemoglobin A1C (Glycated Hemoglobin)

Parameter	Result	Reference range
Hemoglobin	12.2 g/dL	12.0 – 16.0
White blood cells	6.1 ×10⁹/L	4.0 – 10.0
Platelets	245 ×10⁹/L	150 – 400
Sodium	140 mmol/L	135 – 145
Potassium	4.2 mmol/L	3.5 – 5.1
Creatinine	0.82 mg/dL	0.5 – 1.1
Urea	34 mg/dL	15 – 50
AST	22 U/L	<35
ALT	19 U/L	<45
ALP	88 U/L	40 – 130
C-reactive protein	2.1 mg/L	<5
ESR	18 mm/h	<30
Vitamin B12	410 pg/mL	200 – 900
Folate	8.6 ng/mL	3 – 17
TSH	1.9 µIU/mL	0.4 – 4.0
Free T4	1.2 ng/dL	0.8 – 1.8
HbA1c	5.0 %	<5.7

Brain computed tomography did not reveal acute or structural abnormalities.

Subsequently, brain MRI demonstrated marked midbrain atrophy with relative preservation of pontine structures. These findings produced the Mickey Mouse sign (Figure 1) and the hummingbird sign (Figure 2), both supportive of PSP in the appropriate clinical context [3,5].

**Figure 1:**
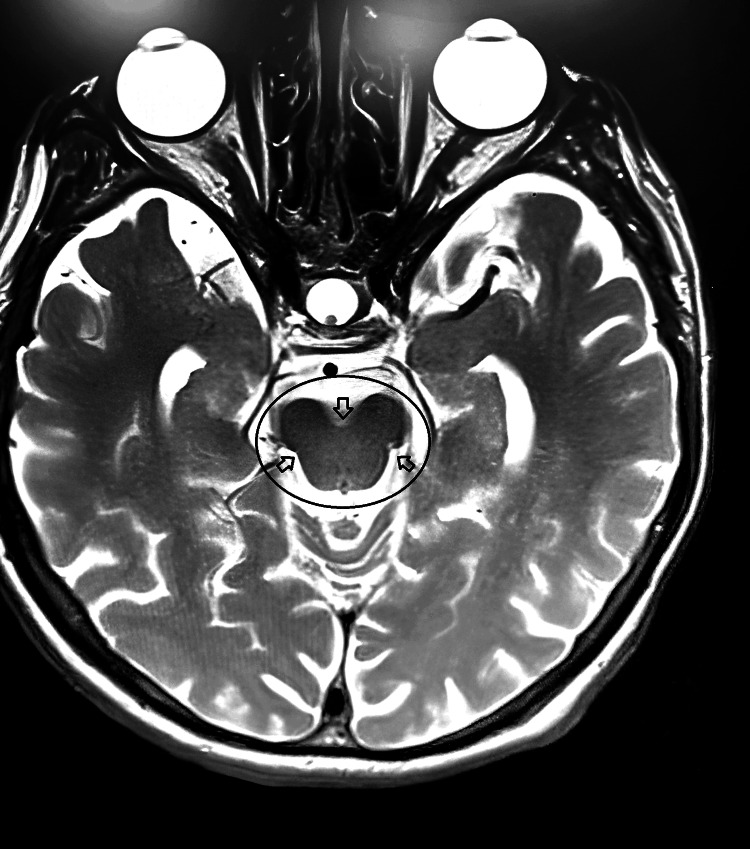
Mickey Mouse sign in progressive supranuclear palsy Axial T1-weighted brain MRI showing selective midbrain atrophy with relative preservation of the cerebral peduncles, resulting in the characteristic “Mickey Mouse sign”. This imaging pattern supports the diagnosis of progressive supranuclear palsy in the appropriate clinical context.

**Figure 2:**
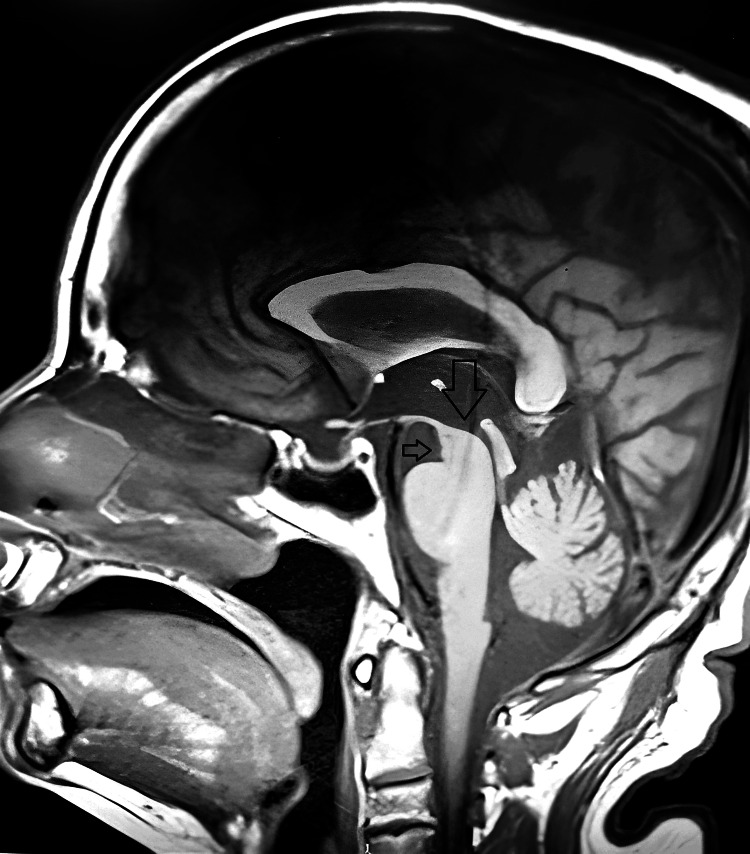
Hummingbird sign in progressive supranuclear palsy Sagittal T1-weighted brain MRI demonstrating marked midbrain atrophy with relative preservation of pontine volume. This structural configuration produces the classic “hummingbird sign”, a neuroimaging feature classically associated with PSP.

DAT-SPECT further demonstrated reduced bilateral putaminal tracer uptake with relative preservation of caudate nuclei uptake. These findings supported the diagnosis of degenerative Parkinsonian syndrome [4].

Based on the clinical presentation, absence of levodopa responsiveness, and characteristic imaging findings, a diagnosis of probable PSP was established according to Movement Disorder Society criteria [1].

The diagnosis was established after repeated neurological evaluations and multidisciplinary discussion with neurologists experienced in atypical Parkinsonian syndromes.

To summarize the temporal evolution of the clinical picture, a chronological reconstruction of the patient’s history is reported in Table 2. 

**Table 2:** Chronological clinical timeline and diagnostic work-up Clinical timeline summarizing the progression from post-COVID systemic decline to rapid neurological deterioration and final diagnosis of probable progressive supranuclear palsy.

Time point	Clinical events
July 2021	Mild SARS-CoV-2 infection with paucisymptomatic course.
Post-COVID (July–September 2021)	Progressive onset of anorexia, dysphagia, and significant weight loss (~20 kg).
September–October 2021	Multiple ENT, endoscopic, and radiological evaluations performed; no structural, neoplastic, or metabolic cause identified.
Early November 2021	Rapid worsening of gait instability and inability to ambulate within 2 weeks.
Few days before admission	Neurological examination revealed axial bradykinesia, hypomimia, hypophonia, reduced stride length, apraxia, and vertical gaze limitation. Initial diagnosis of Parkinsonian syndrome; levodopa therapy initiated without response.
Admission (November 2021)	Hospitalization in Internal Medicine ward for rapid functional decline and diagnostic work-up.
Hospital work-up	Negative oncological, metabolic, and infectious screening. Brain CT normal.
During hospitalization	Brain MRI showed midbrain atrophy with preserved pons (“hummingbird sign” and “Mickey Mouse sign”). DAT-SPECT showed reduced putaminal uptake with preserved caudate nuclei.
Final diagnosis	Probable Progressive Supranuclear Palsy (PSP) according to MDS clinical criteria.
Outcome	Progressive clinical deterioration with loss of autonomy and inability to return home despite optimized supportive care.

During follow-up, the patient showed progressive functional deterioration with persistent severe gait impairment, worsening loss of autonomy, and continued need for supportive care.

## Discussion

Progressive supranuclear palsy is a rare 4-repeat tauopathy characterized by neurodegeneration involving the basal ganglia, brainstem, and cerebellar pathways. Clinically, it typically presents with early postural instability, vertical supranuclear gaze palsy, axial rigidity, dysarthria, dysphagia, and frontal cognitive dysfunction and is associated with a rapidly progressive course and limited therapeutic options [1,2].

In the present case, the initial clinical picture was dominated by nonspecific systemic manifestations, anorexia, dysphagia, and marked weight loss, which preceded overt neurological deterioration. This atypical presentation led to an extensive but unrevealing workup for oncological, metabolic, and structural causes, contributing to diagnostic delay. Such presentations underscore the importance of maintaining a broad differential diagnosis in elderly patients with unexplained functional decline.

Furthermore, the absence of clinical response to levodopa therapy represented an important diagnostic clue, as poor or absent dopaminergic responsiveness remains a hallmark feature distinguishing PSP and other atypical Parkinsonian syndromes from idiopathic Parkinson’s disease [2,8].

The oculomotor findings in this case were characteristic and clinically informative. In addition to vertical supranuclear gaze palsy and slowing of vertical saccades, square wave jerks and eyelid opening apraxia were documented. Square wave jerks, small, involuntary saccadic intrusions during fixation, are frequently observed in PSP and reflect frontal ocular motor dysfunction. Eyelid opening apraxia, characterized by difficulty initiating voluntary eyelid opening in the absence of blepharospasm, is another recognized feature of PSP and may contribute to visual disability [2]. Together, these findings strengthened the clinical diagnosis and reinforced the importance of thorough oculomotor assessment in suspected atypical Parkinsonism.

The diagnostic value of MRI morphometric markers, particularly midbrain atrophy and reduction of the midbrain-to-pons ratio, has been well established and may help differentiate PSP from Parkinson’s disease and multiple system atrophy [5,9]. In addition, DAT-SPECT confirmed presynaptic nigrostriatal degeneration, supporting degenerative Parkinsonian syndrome and aiding differentiation from non-degenerative causes of Parkinsonism [4].

The differential diagnosis included Parkinson’s disease, multiple system atrophy, vascular Parkinsonism, normal pressure hydrocephalus, and structural brainstem lesions. These conditions were excluded based on clinical evolution, neuroimaging findings, and the absence of therapeutic response to levodopa [2,8].

An additional notable aspect of this case is the temporal association between a preceding SARS-CoV-2 infection and the subsequent accelerated functional decline. Although causality cannot be established, systemic infections have been reported to precipitate clinical deterioration in elderly patients with underlying neurodegenerative vulnerability, possibly through mechanisms involving systemic inflammation, metabolic stress, and reduced physiological reserve [6,7]. SARS-CoV-2 infection may have acted as a trigger unmasking a previously subclinical neurodegenerative process; however, this hypothesis remains speculative, and the coincidence of infection and clinical onset does not imply causation.

PSP remains a rapidly progressive disorder with a poor prognosis and no currently available disease-modifying therapies. Management is primarily supportive, and disease progression is typically faster than in Parkinson’s disease, leading to severe functional impairment within a few years [10]. These considerations underscore the importance of early recognition and the integration of clinical, structural, and functional imaging findings to improve diagnostic accuracy.

## Conclusions

This case illustrates the diagnostic complexity of PSP when early manifestations are dominated by nonspecific systemic decline. In elderly patients presenting with unexplained gait impairment, vertical gaze limitation, dysphagia, and poor levodopa responsiveness, PSP should be considered early in the differential diagnosis. A thorough oculomotor examination, including assessment for square wave jerks and eyelid opening apraxia, may provide additional diagnostic value. The integration of clinical evaluation with structural and functional neuroimaging remains crucial for improving diagnostic accuracy and avoiding delayed recognition of atypical Parkinsonian syndromes.
